# Physiological Concentrations of Amyloid Beta Regulate Recycling of Synaptic Vesicles via Alpha7 Acetylcholine Receptor and CDK5/Calcineurin Signaling

**DOI:** 10.3389/fnmol.2017.00221

**Published:** 2017-07-21

**Authors:** Vesna Lazarevic, Sandra Fieńko, Maria Andres-Alonso, Daniela Anni, Daniela Ivanova, Carolina Montenegro-Venegas, Eckart D. Gundelfinger, Michael A. Cousin, Anna Fejtova

**Affiliations:** ^1^RG Presynaptic Plasticity, Leibniz Institute for Neurobiology Magdeburg, Germany; ^2^Department of Neurochemistry and Molecular Biology, Leibniz Institute for Neurobiology Magdeburg, Germany; ^3^German Center for Neurodegenerative Diseases (DZNE) Magdeburg, Germany; ^4^Molecular Psychiatry, Department of Psychiatry and Psychotherapy, University Hospital, University of Erlangen-Nuremberg Erlangen, Germany; ^5^Center for Behavioral Brain Sciences, Otto von Guericke University Magdeburg, Germany; ^6^Medical Faculty, Otto von Guericke University Magdeburg, Germany; ^7^Centre for Integrative Physiology, University of Edinburgh Edinburgh, United Kingdom

**Keywords:** amyloid beta, acetylcholine receptors, synaptic vesicle recycling, neurotransmitter release, CDK5, calcineurin

## Abstract

Despite the central role of amyloid β (Aβ) peptide in the etiopathogenesis of Alzheimer’s disease (AD), its physiological function in healthy brain is still debated. It is well established that elevated levels of Aβ induce synaptic depression and dismantling, connected with neurotoxicity and neuronal loss. Growing evidence suggests a positive regulatory effect of Aβ on synaptic function and cognition; however the exact cellular and molecular correlates are still unclear. In this work, we tested the effect of physiological concentrations of Aβ species of endogenous origin on neurotransmitter release in rat cortical and hippocampal neurons grown in dissociated cultures. Modulation of production and degradation of the endogenous Aβ species as well as applications of the synthetic rodent Aβ_40_ and Aβ_42_ affected efficacy of neurotransmitter release from individual presynapses. Low picomolar Aβ_40_ and Aβ_42_ increased, while Aβ depletion or application of low micromolar concentration decreased synaptic vesicle recycling, showing a hormetic effect of Aβ on neurotransmitter release. These Aβ-mediated modulations required functional alpha7 acetylcholine receptors as well as extracellular and intracellular calcium, involved regulation of CDK5 and calcineurin signaling and increased recycling of synaptic vesicles. These data indicate that Aβ regulates neurotransmitter release from presynapse and suggest that failure of the normal physiological function of Aβ in the fine-tuning of SV cycling could disrupt synaptic function and homeostasis, which would, eventually, lead to cognitive decline and neurodegeneration.

## Introduction

Amyloid beta (Aβ) peptide arises by processing of the extracellular domain of the amyloid precursor protein (APP) mediated by the β-and γ-secretase proteolytic activity. Aβ is famous for its connection with Alzheimer’s disease (AD), the most common form of neurodegeneration, characterized by progressive cognitive decline, memory impairment and formation of amyloid plaques in the brains of affected patients. Aβ is the main component of these plaques (Glenner and Wong, 1984). This fact together with a genetic link of early-onset familiar forms of AD to mutations interfering with the proteolytic processing of APP (Goate et al., 1991) advocate the key role of extracellular Aβ in the pathogenesis of AD. However, the mechanisms by which dysregulated extracellular Aβ disrupts the brain function are still not completely understood. While there is no clear relationship between the number of senile plaques and disease severity, progressive synapse dismantling occurring before formation of any amyloid deposits emerged as the best correlate of the cognitive decline in AD patients and animal models (Terry et al., 1991; Mucke et al., 2000). As synapse loss in AD is preceded by defects in neuronal transmission and plasticity, it has been suggested that extracellular Aβ might be involved in the regulation of these processes (Chapman et al., 1999; Hsia et al., 1999; Walsh et al., 2002; Palop and Mucke, 2010). A large body of evidence supports an inhibitory effect of Aβ on synaptic function. Elevated (high nanomolar and low micromolar) extracellular Aβ reduces neurotransmission mainly by postsynaptic mechanisms including increased internalization or desensitization of postsynaptic glutamate receptors and downstream signaling (Walsh et al., 2002; Hsieh et al., 2006). Recently, it has also been demonstrated that high nanomolar Aβ affects endocytosis of presynaptic neurotransmitter vesicles indicating that the inhibitory effect of Aβ on membrane trafficking is not restricted only to the postsynaptic compartment (Park et al., 2013).

In healthy brain, Aβ is released into the extracellular space depending on neuronal activity (Kamenetz et al., 2003; Cirrito et al., 2005). Thus, due to the widely accepted inhibitory nature of Aβ on the neurotransmission, it has been proposed that endogenous Aβ functions as a negative modulator of synaptic strength in a physiological feed-back mechanism preventing over excitation of brain circuits. Dysregulation of this homeostatic mechanism would trigger synaptic destabilization eventually leading to the development of AD (Palop and Mucke, 2010). However, extracellular concentrations of Aβ in normal brain have been estimated to low picomolar levels, far lower than the concentrations used in most studies showing the Aβ-induced synaptic depression and neurotoxicity (Cirrito et al., 2003; Puzzo et al., 2011). Unexpectedly, several studies investigating the impact of physiological concentration of Aβ revealed a positive effect on neuroplasticity and learning (Puzzo et al., 2008, 2011; Garcia-Osta and Alberini, 2009). Hippocampal long-term potentiation (LTP) and learning were improved upon application of synthetic mouse or human Aβ_42_ in picomolar concentrations (Puzzo et al., 2008, 2011; Garcia-Osta and Alberini, 2009), whereas high nanomolar Aβ, in the same experimental setting, led to well-established reduction of LTP suggesting a hormetic nature of Aβ on synaptic plasticity (Puzzo et al., 2008). Interestingly, these effects were sensitive to α-bungarotoxin, a selective antagonist of α7 nicotinic acetylcholine receptor (α7nAChR), and absent in α7nAChR knockout mice, which implies that functional α7nAChRs are required for Aβ_42_-induced neuroplasticity (Puzzo et al., 2008, 2011). This is consistent with the reported high-affinity binding of Aβ to α7nAChR (Wang et al., 2000a,b) and increased calcium influx through α7nAChRs in isolated hippocampal synaptosomes upon application of picomolar Aβ_42_ (Dougherty et al., 2003). It has been proposed that the positive impact of Aβ_42_ on neurotransmission is mediated by potentiating neurotransmitter release from presynapse (Puzzo et al., 2008; Abramov et al., 2009). Abramov et al. (2009) convincingly demonstrated modulation of presynaptic release probability in rat and mice hippocampal neuronal cultures treated with thiorphan (Th), an inhibitor of the rate-limiting peptidase neprilysin involved in the extracellular clearance of Aβ species. Later on, the same laboratory described the modulation of presynaptic release probability in the same rodent cultures upon application of picomolar amounts of human Aβ_1–40_ peptide (Abramov et al., 2009; Fogel et al., 2014). In contrast to *ex vivo* electrophysiological experiments in hippocampal slices and behavioral analyses (Puzzo et al., 2008, 2011), both studies in cultured cells argued against the contribution of α7nAChR to the effect of Th or Aβ_1–40_ on neurotransmission and proposed an alternative pathway involving APP homodimerization and signaling via heteromeric G_i/o_ proteins (Fogel et al., 2014).

Thus, it is unclear, whether different species of endogenous Aβ peptides exert the same effect on presynapse, what is the contribution of α7nAChRs, and what signaling connects putative Aβ receptors to the regulation of neurotransmitter release form presynapse. To address these questions, we tested systematically presynaptic effects of Th and rodent Aβ_1–40_ as well as Aβ_1–42_ in low to intermediate picomolar and low micromolar concentrations in cultured cortical neurons. To this end we visualized and quantified synaptic vesicle (SV) recycling within individual presynaptic boutons in living cells and investigated the contribution of α7nAChRs and their downstream signaling to the Aβ-mediated regulation of presynaptic function. Our data have potential implications for the pathophysiology of AD. Since Aβ modulates neurotransmission at very low extracellular concentrations, this physiological function would be directly affected already upon minor changes in extracellular Aβ levels occurring in early phases of AD and thus might contribute to cognitive impairments far before formation of amyloid plaques.

## Materials and Methods

### Antibodies

For immunocytochemical stainings (ICC) and for Western blots (WB) following primary antibodies were used from rabbit: anti-CDK5 (WB 1:1000, C-8 Santa Cruz), anti-homer1 (ICC 1:1000, Synaptic Systems), anti-VGLUT1 (ICC 1:1000, Synaptic Systems), anti-VGAT (ICC 1:1000, Synaptic Systems), anti-VGAT lumenal domain Oyster550-labeled (ICC 1:200, Synaptic Systems), from mouse: anti-synaptotagmin1 lumenal domain Oyster550-labeled (ICC 1:250, Synaptic Systems), anti-β-tubulin isotype III (WB 1:2000, Sigma), anti-Aβ_17–24_ (4G8) (5 μg/ml, Signet), and from guinea pig: anti-synaptophysin (ICC 1:1000, Synaptic Systems). For ICC Alexa Fluor 488- (1:2000), Cy3- (1:2000) and Cy5- (1:1000) fluorescently labeled secondary antibodies were purchased from Jackson ImmunoResearch. For WB secondary antibodies labeled with Alexa Fluor 680 (1:20,000, ThermoFisher Scientific/Molecular Probes) and IRDye 800CW (1:20,000, Rockland) were used.

### Chemical Reagents

Thiorphan (Th), FK-506 monohydrate, TMB8 and Choline chloride were purchased from Sigma-Aldrich. β-Secretase inhibitor IV, InSolution γ-Secretase inhibitor L-685, 458, InSolution Roscovitine, α-Bungarotoxin and Bafilomycin A1 from Calbiochem. Aβ_1–42_ and Aβ_1–40_ peptides, D-(-)-2-Amino-5-phosphonopentanoic acid (APV), 6-Cyano-7-nitroquinoxaline-2,3-dione disodium (CNQX), BAPTA-AM and PNU 120596 from Tocris. Aβ was diluted according to the manufacturer’s instruction. Th was diluted to 1 mM stock solution in artificial cerebrospinal fluid (ASCF) supplemented with 1 mM ascorbic acid to prevent Th oxidation (Iwata et al., 2001; Abramov et al., 2009). In all experiments control cells were treated with ascorbic acid in ASCF.

### Animals

Breeding of animals and experiments using animal material were carried out in accordance with the European Communities Council Directive (2010/63/EU) and approved by the local animal care committees of Sachsen-Anhalt and Middle-Franconia/Germany.

### Primary Neuronal Cultures

Primary cultures of cortical neurons were prepared as described previously (Lazarevic et al., 2011). In brief, rat embryos at day 18–19 after fertilization (E18–E19) were sacrificed by decapitation. The brains were removed and deprived of meninges. After treatment with 0.25% trypsin for 15 min and mechanical trituration cell suspension was plated in DMEM containing 10% fetal calf serum (FCS), 1 mM glutamine and antibiotics (100 U/ml penicillin, 100 μg/ml streptomycin) onto poly-D-lysine coated glass coverslips (Sigma, 18 mm diameter). Twenty-four hours after plating, the medium was exchanged for Neurobasal medium supplemented with B27 (Life Technologies), antibiotics, and 0.8 mM glutamine. The cells were maintained in a humidified incubator with 5% CO_2_. Primary hippocampal cultures were prepared according to a modified original protocol from Banker (1980) as described in Frischknecht et al. (2008). Briefly, rat embryos were sacrificed at E18–E19, brains were removed, hippocampi extracted and subjected to trypsin digestion and mechanical trituration. Thereafter, cells suspended in DMEM containing 10% FCS, 1 mM glutamine and penicillin/streptomycin were plated onto poly-L-lysine-coated glass coverslips. After 1 h, coverslips with primary hippocampal neurons were transferred into a Petri dish containing an astrocytic monolayer in Neurobasal medium supplemented with B27, antibiotics and glutamine, as described before, and placed in a humidified incubator. Neurons cultured for 18–21 days *in vitro* (DIV) were used for all analyses. For immunocytochemistry and Aβ_42_ ELISA cells were plated on poly-D-lysine-coated glass coverslips at a density of 50,000 cells/12 mm coverslip in 24-well plates in 0.5 ml growth media. Aβ_40_ ELISA was done on cells plated at density 100,000 cells/18 mm coverslips in 12-well plates in 1 ml of growth media. For imaging experiments cells were plated on poly-L-lysine-coated glass coverslips at a density of 30,000 cells/18 mm coverslip in a 60-mm Petri dish. For biochemical experiments cells were plated in 6-well plates at a density of 300,000 cells/well.

### ELISA Measurements of Aβ

The concentration of Aβ peptides in extracellular medium was assessed by sandwich ELISA using Human/Rat Aβ(42) high sensitive kit and Human/Rat Aβ(40) kit II purchased from Wako. The measurements were done according to manufacturer’s protocol always using fresh medium.

### Immunocytochemistry and Synaptotagmin1 Luminal Domain Antibody Uptake

Neurons were fixed with 4% paraformaldehyde, 4% sucrose in PBS pH 7.4, for 3 min at RT. Prior to immunostaining, the cells were blocked and permeabilized with PBS containing 10% FCS, 0.1% glycine and 0.3% TritonX-100 for 30 min. Subsequently, primary antibodies were applied overnight at 4°C. After three washing steps with PBS at RT coverslips were incubated with secondary antibodies for 1 h at RT. Both, primary and secondary antibodies were diluted in PBS containing 3% FCS. Coverslips were mounted on slides with Mowiol (Calbiochem) and kept at 4°C until microscopic analysis. Synaptotagmin1 luminal domain antibody uptake was done as described (Lazarevic et al., 2011). Cells were briefly washed with freshly prepared Tyrode’s buffer (119 mM NaCl, 2.5 mM KCl, 2 mM CaCl_2_, 2 mM MgCl_2_, 30 mM glucose, 25 mM HEPES pH 7.4) and incubated with fluorescently-labeled syt1 antibody diluted in the same buffer, either for 20 min at 37°C to monitor network activity driven uptake, or for 4 min at RT in Tyrode’s buffer containing 50 mM KCl and 71.5 mM NaCl to assess evoked syt1 Ab uptake. Thereafter, samples were fixed and stained. In each experiment, at least two coverslips per treatment were processed in parallel. The results are representative of 2–5 independent experiments.

### Lentiviral Particles Production

The original mRFP-synaptophysin-pHluorin (sypHy) construct was obtained T. Oertner (Rose et al., 2013). cDNA of SypHy was inserted into FUGW backbone vector by standard cloning. Lentiviral particles were generated in HEK293T cell line (ATTC, Manassas, VA, USA) using FUGW-based transfer, psPAX2 packaging and pVSVG pseudotyping vectors (Lois et al., 2002). HEK293T cells were grown in media containing 10% FCS to 60% confluence in 75 cm^2^ flasks. The cells were transfected using the calcium phosphate method (Fejtova et al., 2009). Molar ratio of FUGW: psPAX2: pVSVG was 2:1:1. Twenty-four hours later the content of FCS was reduced to 4%. After 48 h, the virus-containing media was collected and cleared from large cellular debris by centrifugation for 20 min at 2000 *g*. The supernatant was aliquoted and stored at −80°C until further use.

### Synapto-pHluorin Imaging

SypHy was delivered to rat hippocampal neurons cultured for 4 DIV by the means of lentiviral infection and subjected to imaging at DIV17-18. Coverslips were incubated with Aβ_1–42_ or water as a control in a conditioned media for 1 h in the incubator. Coverslips were installed in an imaging chamber (Warner Instruments) and imaged at RT on an inverted microscope (Observer. D1; Zeiss) endowed with an EMCCD camera (Evolve 512; Delta Photometrics) controlled by MetaMorph Imaging (MDS Analytical Technologies). A 63× objective and GFP/mCherry single band exciters ET filter set (exciter 470/40, exciter 572/35, emitter 59022m, dichroic 59022BS) were used. Transduced neurons were identified by RFP expression. Neurons were stimulated in the presence of bafilomycin A1 (1 μM) to prevent vesicle reacidification and APV (50 μM) and CNQX (10 μM) to block recurrent network activity with 40 AP at 20 Hz, followed by 2 min recovery period. Afterwards, a stimulation with 900 AP at 20 Hz was delivered and a pulse of 60 mM NH4Cl-containing solution applied (Burrone et al., 2006). Electrical stimulation was delivered by a S48 stimulator unit (GRASS Technologies). A stream of images was acquired at 10 Hz and 5 s of the baseline was recorded before each stimulation, followed by imaging of the recovery phase for another 60 s. Synaptic boutons responsive to stimulation were selected by subtracting the first 10 frames of the baseline (before stimulation) from the 10 frames directly after the stimulus. Only neurons showing ≤20% increase in the fluorescence after NH_4_Cl application were considered as viable and metabolically active and included for the analysis. The mean IF intensities were measured in the circular regions of interest (ROIs with a diameter of 8 × 8 pixel) placed over each responding synapse using Time Series Analyzer V2.0 plugin in ImageJ and plotted after bleaching correction using GraphPad Software. The relative sizes of the RRP and the RP were expressed as fractions of the total sypHy-expressing pool detected after addition of NH_4_Cl. RP was quantified by averaging the mean of 50 values per each cell (representative of the frames 390–440 corresponding to time points 39–44 s on the XY graph). The results are representative of 3–5 independent experiments.

### Image Acquisition and Analysis

Images were acquired with Zeiss Axio Imager A2 microscope with Cool Snap EZ camera (Visitron Systems) and MetaMorph Imaging software (MDS Analytical Technologies). For each pair of coverslips (treated vs. control) the same exposure time was taken. Per each experimental condition two coverslips were individually treated and processed. Images were captured from at least 3–5 visual fields (= cells) per coverslip and further analyzed using NIH ImageJ and OpenView software (Tsuriel et al., 2006). Upon appropriate background subtraction, immunoreactive puncta were counted along the 20 μm of proximal (≥10 μm and ≤50 μm distance from the cell body) or distal dendrite (≥50 μm distance from the cell body). The synaptic immunofluorescence intensities (IF) were assessed in a region of interest (ROI) set by the mask in the channel for synaptophysin (sph), which was used as synaptic marker. The mask was created semiautomatically using OpenView software.

### Quantitative Western Blot

Control and treated 3 weeks old cortical neurons were washed with ice cold PBS and lysed in lysis buffer (50 mM Tris pH 7.5, 150 mM NaCl, 1 mM EDTA, 1 mM EGTA) supplemented with Complete protease inhibitors (Roche), phosphatase inhibitor cocktail PhosStop (Roche) and Calpain inhibitor PD150606 (Tocris). Precleared cell lysates were mixed with SDS loading buffer. Equal amount of proteins was loaded on SDS-PAGE and electrotransferred to Millipore Immobilon-FL PVDF membranes. Membranes were incubated over night at 4°C with primary antibodies and 1 h at RT with fluorescently labeled secondary antibody. Immunodetection and quantification was carried out using Odyssey Infrared Imagine System and Odyssey software v2.1 (LI-COR). After appropriate background subtraction all values were normalized using βIII-tubulin as a loading control.

### CDK5 Immunoprecipitation and Activity Assay

The kinase assay was performed as described in Crews et al. (2011). Briefly, 3 weeks old cortical neurons were washed with ice cold PBS and lysed in CDK5 IP buffer (50 mM Tris pH 7.5, 150 mM NaCl, 1 mM EDTA, 1 mM EGTA) supplemented with Complete protease inhibitors, phosphatase inhibitor cocktail PhosStop and Calpain inhibitor PD150606. Precleared cell lysates were subjected to immunoprecipitation using GammaBind Plus Sepharose beads (GE Healthcare) coupled with rabbit polyclonal antibody against CDK5 (C-8; Santa Cruz Biotechnology). After 3 h incubation at 4°C, immunoprecipitates were washed three times with CDK5 IP buffer and resuspended in 50 μl of CDK5 kinase buffer (25 mM Tris pH 7.5, 10 mM MgCl_2_) in the presence of 90 μM ATP and 0.1 mM CDK5 substrate, Histone H1 (PKTPKKAKKL; sc-3066 Santa Cruz). Samples were then incubated for 30 min at 30°C and the reaction was stopped by adding 50 μl of Kinase glo plus reagent (Promega). Luminescent signal was measured on FLUOstar Omega microplate reader (BMG Labtech).

### Calcineurin Activity Assay

Calcineurin activity was assessed using calcineurin cellular activity assay kit (Calbiochem, Cat. No. 207007) according to the manufacturer’s instructions. In brief, 3 weeks old primary cortical neurons were lysed in the buffer supplied by the manufacturer (25 mM Tris-HCl pH 7.5, 0.5 mM dithiothreitol, 50 μM EDTA, 50 μM EGTA, 0.2% Nonidet P-40). Upon removal of free phosphate samples were incubated with calcineurin substrate, RII phosphopeptide (DLDVPIPGRFDRRVpSVAAE), in the assay buffer containing 100 mM NaCl, 50 mM Tris-HCl (pH 7.5), 6 mM MgCl_2_, 0.5 mM CaCl_2_, 0.5 mM dithiothreitol, 0.05% Nonidet P-40. After 30 min incubation at 30°C, reactions were terminated by adding 100 μl GREEN reagent. A_620_ was measured using a microtiter plate reader. After background subtraction from each sample, the activity of calcineurin was determined as the difference between total phosphatase activity and the phosphatase activity in the EGTA containing buffer.

### Statistics

All statistical analyses were performed with Prism 5 software (GraphPad Software). The normal distribution of the data was assessed using D’Agostino-Pearson omnibus test. Accordingly, parametric or non-parametric test was applied (as indicated in each experiment). In all graphs numbers within bars depict the numbers of analyzed individual visual fields (= cells) or independent wells/samples obtained from at least two different cell culture preparations. All data are always normalized to the mean of the control group and expressed as mean ± SEM. The level of statistical significance is indicated as *p < 0.05, **p < 0.01, ***p < 0.001 in all the graphs.

## Results

### Th Modulates Recycling of SVs at Excitatory and Inhibitory Synapses

**Figure 1:**
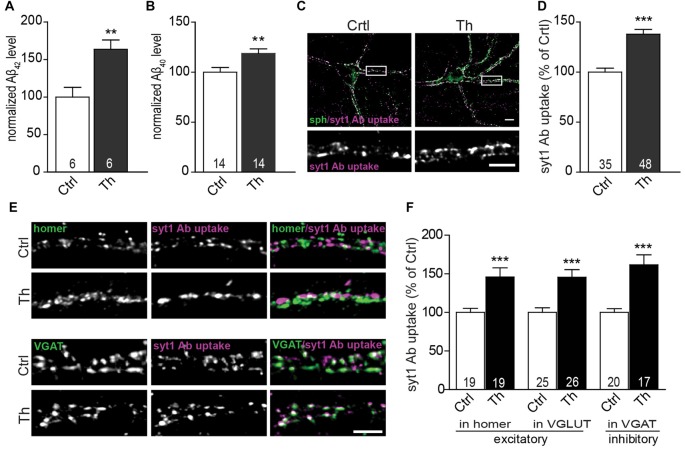
Endogenous Aβ modulates presynaptic activity at excitatory and inhibitory synapses. **(A,B)** Quantification of Aβ_x-42_ **(A)** and Aβ_x-40_ **(B)** levels in the medium from vehicle- and Th-treated cortical cells using sandwich ELISA. Numbers within columns represent the number of individually treated and analyzed wells obtained from two **(A)** and three **(B)** independently prepared cell cultures. **(C)** Representative images of syt1 Ab uptake (magenta) from vehicle- and Th-treated cortical neurons. Synaptophysin (sph, green) was used as a synaptic marker. Regions showed in high magnification are boxed. **(D)** Quantification of normalized fluorescence intensity of network activity-driven syt1 Ab uptake in control and Th-treated neurons along proximal dendrites. **(E)** Representative images of syt1 Ab uptake, from cortical neurons incubated with Th or control solution, at excitatory (homer, green) and inhibitory (VGAT, green) terminals. **(F)** Statistical quantification of the intensity of syt1 Ab uptake in puncta positive for either excitatory (homer, VGLUT) or inhibitory (VGAT) synapses driven by the spontaneous network activity. Numbers within columns show the number of analyzed cells derived from five **(D)** and two **(F)** independent cultures. Values are expressed as the mean ± SEM. Effect of treatment was compared to untreated control by unpaired two-tailed Student *t* test (**A,B,D,F** syt1 Ab uptake in VGLUT-positive and VGAT-positive) or Mann Whitney test (**F**, syt1 Ab uptake in homer-positive puncta); **p < 0.01, ***p < 0.001. Scale bars represent 10 μm in overview and 5 μm in insets.

In order to investigate the role of endogenously secreted Aβ in the regulation of neurotransmitter release, we applied Th to the cultured rat cortical neurons at DIV18-21. Th is an inhibitor of metalloprotease neprilysin, which mediates the rate-limiting step in Aβ degradation (Iwata et al., 2001; Abramov et al., 2009). Treatment with Th for 1 h led to 1.6 and 1.2 fold elevation in the extracellular concentrations of Aβ_42_ and Aβ_40_, respectively compared to untreated control, as assessed by sandwich ELISA of freshly collected neuronal growth media (Figure 1A; [Aβ_42_]: control: 57 ± 11 pM vs. Th: 94 ± 15 pM, Figure 1B; [Aβ_40_]: control: 130 ± 11 pM vs. Th: 153 ± 11 pM). Low pM concentrations of endogenously produced Aβ_42_ and Aβ_40_ peptides measured here as well as their Th-mediated elevation are in line with previous reports and comparable with concentrations obtained *in vivo* (Cirrito et al., 2005; Abramov et al., 2009).

To examine the effect of Th on presynaptic function, we monitored the efficacy of SV recycling at the level of individual synapses using synaptotagmin1 antibody (syt1 Ab) uptake (Kraszewski et al., 1995; Lazarevic et al., 2011). This assay utilizes a fluorophore-labeled antibody that recognizes a luminal domain of the SV protein syt1. This epitope becomes accessible for the antibody, added to neuronal media, only after fusion of SVs for neurotransmitter release before they undergo rapid compensatory endocytosis. Network activity-driven syt1 Ab uptake was significantly increased in cultures treated with Th compared to untreated control (Figures 1C,D; Th 138 ± 5% of control). Interestingly, application of Th led to an increased network activity-driven recycling of SV in both excitatory and inhibitory synapses as shown by quantification of syt1 Ab uptake in puncta immunoreactive for glutamatergic markers homer and vesicular glutamate transporter 1 (VGLUT1) or for a marker of inhibitory synapses, vesicular GABA transporter (VGAT; Figures 1E,F; Homer, Th: 146% ± 12%; VGLUT1, Th: 145 ± 10%; VGAT, Th: 162 ± 13% of control). Th-mediated increase of SV recycling in inhibitory synapses was further confirmed by performing uptake assay with an antibody against luminal domain of VGAT (Th: 151 ± 9% of control).

### Extracellular Aβ_40_ and Aβ_42_ Exert Hormetic Effect on SV Recycling

**Figure 2:**
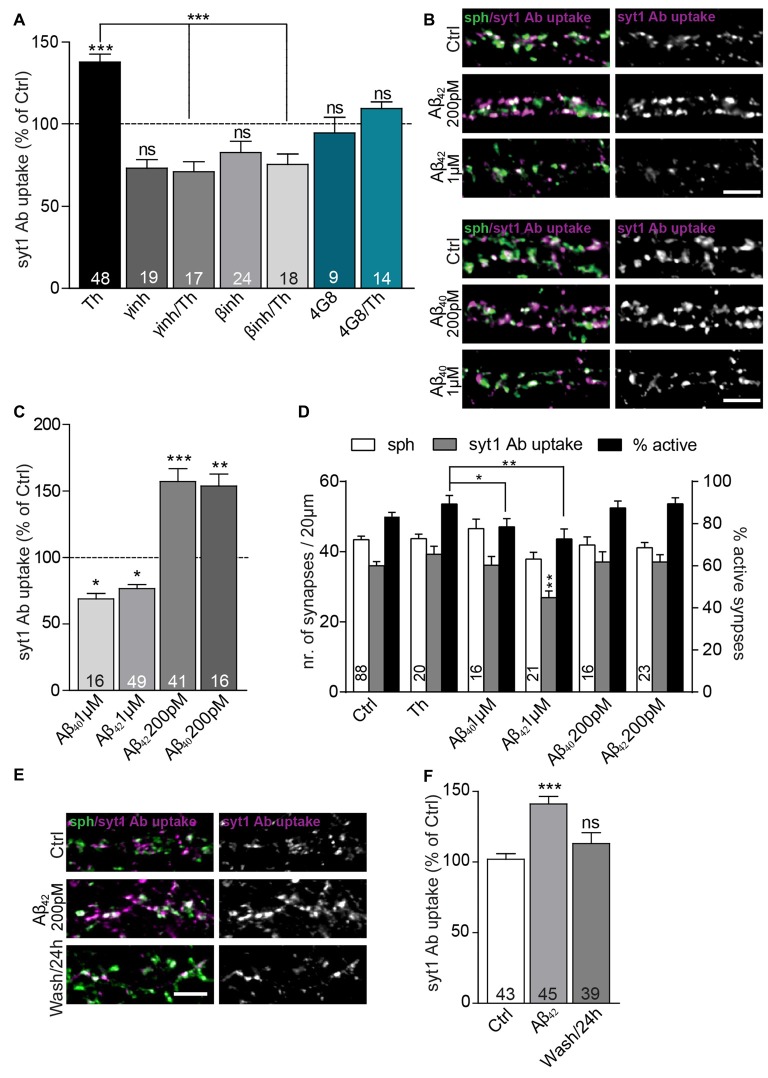
Extracellular Aβ_40_ and Aβ_42_ have a hormetic effect on presynaptic vesicle recycling. **(A)** Quantification of activity-driven syt1 Ab uptake from Th-treated vs. control cells in the presence or absence of Aβ antibody (4G8) and inhibitors of enzymes involved in amyloid precursor protein (APP) processing (β-secretase and γ-secretase inhibitors). Numbers within bars display the number of analyzed visual fields pooled from five (Th), three (βinh; γinh) and two (4G8) independent cell culture preparations. **(B)** Representative images of cultured cortical neurons treated either with pM or μM concentration of Aβ_40_, Aβ_42_ or control solution and stained with syt1 Ab (magenta) and sph (green). **(C)** Quantification of **(B)**. Numbers indicate the number of individual cells used for analysis obtained from four (Aβ_42_) and two (Aβ_40_) culture preparation.** (D)** Quantification of density of sph-positive synapses (white bars), active synapses with syt1 Ab uptake (gray bars) and quantification of percentage of active synapses (black bars) in cultures treated with vehicle, Th and 200 pM and 1 μM Aβ_40_ and Aβ_42_. Numbers in columns indicate number of analyzed cells coming from at least two independent cell preparations. **(E)** Representative images of cortical neurons treated with control or 200 pM Aβ_42_ as well as cells assayed 23 h after Aβ_42_ washout for network activity-driven syt1 Ab uptake. **(F)** Quantification of the experiment in **(E)**. Numbers within columns show the number of the cells used for analysis from at least two independent cell culture preparations. In all graphs values are expressed as the mean ± SEM. The density of synapses were analyzed using one-way ANOVA with Bonferroni *post hoc* test, all other data were analyzed using Kruskal-Wallis test followed by Dunn’s multiple comparison test, *p < 0.05, **p < 0.01, ***p < 0.001. Scale bar, 5 μm.

To confirm that Th-induced changes in SV recycling rely on the modulation of the extracellular concentration of the endogenously secreted Aβ peptides, we treated neurons with Th in the presence or absence of 4G8 monoclonal antibody (5 μg/ml) that specifically binds Aβ peptides. While incubation with Th alone clearly increased SV recycling, this effect was completely prevented by chelation of extracellular Aβ using 4G8 antibody (Figure 2A; Th: 138 ± 5%; 4G8/Th: 110 ± 4% of control). Next, we inhibited production of Aβ, either by blocking β-secretase (BACE inhibitor IV, 0.5 μM) or γ-secretase (GAMMAinh, L-685, 458, 0.2 μM). Treatment with these inhibitors for 6 h led to a notable decrease of the syt1 Ab uptake, pointing to the role of endogenously released Aβ in the modulation of basal SV recycling (Figure 2A; βinh: 83 ± 7%; γinh: 73 ± 5% of control). Moreover, pre-treatment with secretase inhibitors, 5 h prior to the treatment with Th for 1 h, fully blocked the Th-induced increase in SV recycling (βinh/Th: 76 ± 6%; γinh/Th: 71 ± 6% of control). Taken together these experiments strongly support involvement of endogenously secreted Aβ in the Th-induced increase in the syt1 Ab uptake.

To strengthen this assumption, we added synthetic Aβ_42_ and Aβ_40,_ in physiological concentrations of 200 pM, to the growth media for 1 h, which caused a substantial increase in the syt1 Ab uptake (Figures 2B,C; Aβ_40_: 154 ± 9%; Aβ_42_: 157 ± 9% of control). In contrary, 1-h treatment with peptides from identical preparation at 1 μM concentration, widely used to induce neurotoxic effects (Park et al., 2013), had an opposite effect and led to a significant decrease in syt1 Ab uptake (Figures 2B,C; Aβ_40_: 69 ± 4%; Aβ_42_: 77 ± 3% of control). None of the treatments affected the density of synapses contacting proximal dendrites assessed as number of puncta immunoreactive for synaptic vesicle protein sph (Figure 2D; control: 43 ± 1; Th: 43 ± 1; 200 pM Aβ_40_: 42 ± 2, Aβ_42_: 41 ± 1; 1 μM Aβ_40_: 47 ± 3, Aβ_42_ 38 ± 2 synapses). The number of active synapses assessed as sph puncta with over-threshold immunofluorescence for syt1 Ab uptake was decreased in cells treated with 1 μM Aβ_42_ but unchanged in all other conditions (Figure 2D, control: 36 ± 1; Th: 39 ± 2; 200 pM Aβ_40_: 37 ± 3, Aβ_42_: 37 ± 2; 1 μM Aβ_40_: 36 ± 3, Aβ_42_ 27 ± 2 active synapses). The percentage of active synapses calculated as proportion of active synapses out of sph positive synapses for each analyzed visual field differed between Th-treated cells and cells treated with 1 μM Aβ peptides further indicating that elevated physiological and supraphysiological concentrations of Aβ_40_ and Aβ_42_ exert opposite effects on presynaptic function (Figure 2D, control: 83 ± 2%; Th: 89 ± 4%; 200 pM Aβ_40_: 87 ± 3%, Aβ_42_: 89 ± 3%; 1 μM Aβ_40_: 79 ± 4%, Aβ_42_ 73 ± 5%). These results are in line with a hormetic effect of Aβ_40_ and Aβ_42_ peptides, with low, physiological (high pM) concentration potentiating SV recycling, and high, supraphysiogical (low μM) concentration having a negative impact in the identical experimental readout.

To study durability and reversibility of Aβ-mediated effect on SV recycling, cells were treated with 200 pM Aβ_42_ for 1 h prior to the washout and replacement with the conditioned medium. In this experiment, cells monitored 1 h after 200 pM Aβ_42_ treatment showed a significant increase in syt1 Ab uptake. In contrast, cells assayed 23 h after the Aβ_42_ washout showed recycling indistinguishable from untreated controls (Figures 2E,F; Aβ_42_: 141 ± 5%; Aβ_42_/washout: 113 ± 8% of control). This suggests that modulation of Aβ production and clearance might represent a physiological mechanism, inducing rapid changes in the recycling of SVs.

### Aβ Potentiates Basal Neurotransmission via an Increase in the Recycling Pool of SVs

**Figure 3:**
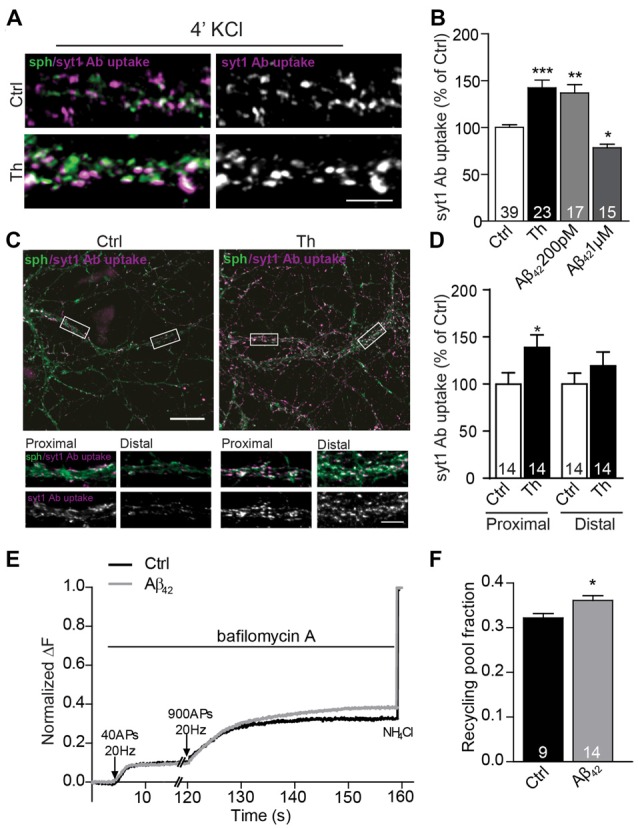
Picomolar Aβ increases the size of the functional recycling pool. **(A)** Representative images of depolarization-induced syt1 Ab uptake in control and Th-treated neurons. **(B)** Quantification of evoked syt1 Ab uptake after application of Th and pM or μM Aβ_42_. Numbers represent the number of cells used for analyses derived from three (Th) and two (Aβ) independent cultures. ** (C)** Representative images of syt1 Ab uptake from control or Th-treated hippocampal neurons contacting proximal as well as distal dendritic segments. Proximal and distal areas where the boutons were analyzed are boxed. **(D)** Quantification of the data in **(C)**. Values within columns correspond to the number of analyzed visual fields pooled from two independent cultures.** (E)** Averaged traces from hippocampal cells expressing sypHy and treated with control or 200 pM Aβ_42_. Intensities are normalized to the maximal NH_4_Cl response. **(F)** Mean values of the recycling pool. Numbers within columns represent the number of the analyzed cells from at least three independent cell culture preparations. Values are expressed as the mean ± SEM. Statistical significance was assessed by Kruskal-Wallis test followed by Dunn’s multiple comparison test **(B)**, unpaired two-tailed Student *t* test **(D)** or Mann Whitney test **(F)** *p < 0.05, **p < 0.01, ***p < 0.001. Scale bars are10 μm in overview and 5 μm in insets.

The observed increase in the network activity-driven SV recycling points to changes in presynaptic properties, but could also reflect elevated network activity in Th- or Aβ-treated cultures. To test whether this effect relies on a modulation of presynaptic mechanisms, we performed syt1 Ab uptake during pulse-application of 50 mM KCl, which depolarizes neuronal membranes and induces release of all releasable SVs, i.e., recycling pool (RP) of SVs (Alabi and Tsien, 2012). Depolarization-induced syt1 Ab uptake was upregulated by 40% in Th-treated cultures (Figures 3A,B; Th: 142 ± 8% of control) suggesting the role of the endogenous Aβ in the regulation of the recycling pool of SV. Accordingly, application of 200 pM Aβ_42_ exerted similar effect (Figure 3B; Aβ_42_: 137 ± 9% of control). Interestingly, high 1 μM concentration of synthetic Aβ_42_ significantly reduced the depolarization–induced recycling confirming a hormetic nature of Aβ-mediated modulation of the presynaptic properties (Figure 3B; Aβ_42_: 78 ± 4% of control).

Since syt1 Ab uptake enables only end point measurement of SV recycling and fully relies on the endogenous expression of syt1, we sought to assess whether SV turnover was increased by an alternative method. To this end, we moved to hippocampal neurons, where the potentiating effect of Th, Aβ_40_ and Aβ_42_ was originally described by others and reproduced by us in the synapses contacting proximal dendritic segments (Figures 3C,D; Th proximal: 139 ± 13% of proximal control; Th distal: 119 ± 15% of distal control). We performed live imaging of SV turnover implementing genetically encoded pH-sensitive probe called synaptophysin-pHluorin (sypHy; Burrone et al., 2006; Granseth et al., 2006; Rose et al., 2013) expressed in cultured hippocampal neurons using lentiviral vectors. SypHy fluorescence is quenched at low, acidic pH found inside the SVs, but increases upon SV fusion and exposure of their lumen to the neutral pH of the extracellular media. The imaging was performed in the presence of bafilomycin, an inhibitor of the vesicular proton pump that prevents reacidification of SVs upon their compensatory endocytosis. To visualize readily releasable pool (RRP), which corresponds to the morphologically docked SVs, we delivered 40 AP at 20 Hz (Burrone et al., 2006). After 2 min pause to allow for the recovery of RRP, 900 AP at 20 Hz were delivered to release all releasable SVs (RP). SV refractory to stimulation (resting pool, RtP) were visualized by the alkalization of the SVs lumen using NH_4_Cl pulse, which enables accurate assessment of RP relative to all SVs present at the individual synapse (Burrone et al., 2006). In neurons treated with Aβ_42_ no changes in RRP, but a significant increase of RP (Figures 3E,F; Aβ_42_: 112 ± 1% of control) and correspondingly a decrease in RtP were observed. These experiments ultimately demonstrate that Aβ modifies presynaptic function by the regulation of the turnover of presynaptic SV.

### Role of Calcium Signaling via α7nAChR in the Aβ-Mediated Potentiation of SV Recycling

**Figure 4:**
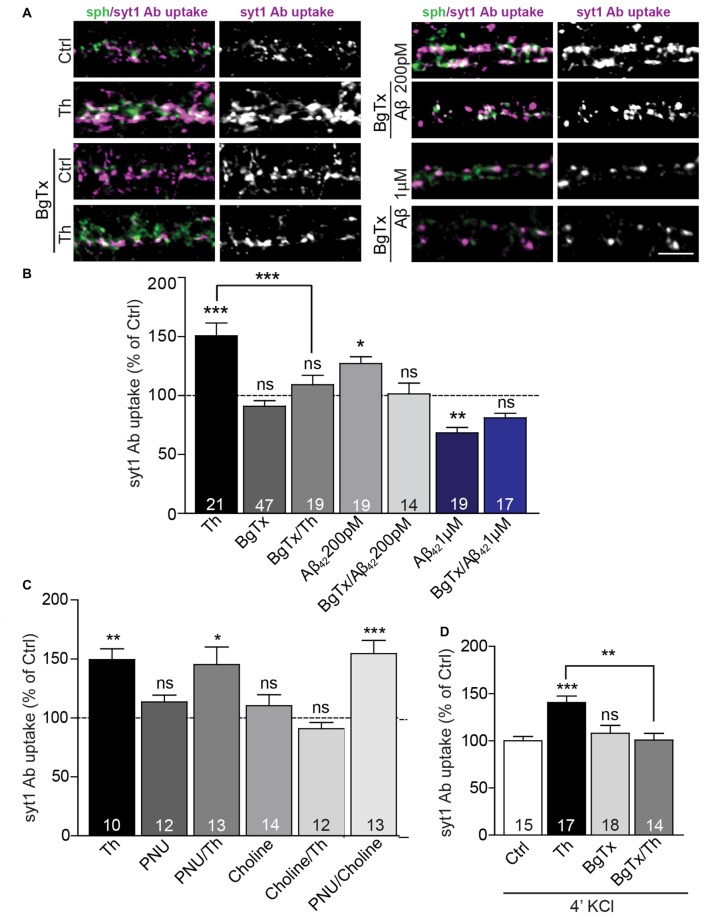
Picomolar Aβ acts via α7nACh receptors. **(A)** Representative images of syt1 Ab uptake from Th-, Aβ_42_ 200 pM or Aβ_42_ 1 μM-treated neurons pre-incubated with BgTx to investigate the effect of α7nAChR. **(B)** Quantification of syt1 Ab uptake from **(A)**. Numbers within columns indicate numbers of analyzed cells from two (Th; Aβ_42_ 200 pM) or three (Aβ_42_ 1 μM) cell culture preparations. **(C)** Statistical analysis of syt1 Ab uptake from cortical neurons treated with control, Th or/and ortho- and allosteric modulators of α7nACh receptors. **(D)** Quantification of depolarization-induced syt1 Ab uptake after control, Th and/or BgTx application. The numbers within bars **(C,D)** indicate the number of analyzed cells obtained from two separate cell culture preparations. In all graphs the values are expressed as the mean ± SEM. Statistical significance was evaluated by Kruskal-Wallis test followed by Dunn’s multiple comparison test **(B,C)** or one-way ANOVA with Bonferroni *post hoc* test **(D)**; *p < 0.05, **p < 0.01, ***p < 0.001. Scale bar 5 μm.

Previous studies proposed that, at picomolar concentrations, Aβ could bind and activate presynaptic α7nAChR (Wang et al., 2000b; Dougherty et al., 2003). Thus, we sought to determine the role of these receptors in the Aβ-mediated regulation of the presynaptic neurotransmitter release. Treatment with α-bungarotoxin (BgTx, 50 nM), a specific blocker of α7nAChRs, for 90 min did not significantly affect syt1 Ab uptake in untreated cells. However, the same treatment completely prevented increase in syt1 Ab uptake induced by Th- or 200 pM Aβ_42_ application for 1 h, revealing a critical role of α7nAChR in the Aβ_42_-mediated regulation of SV cycling (Figures 4A,B; BgTx: 91 ± 5%; Th: 151 ± 11%; BgTx/Th: 109 ± 8%; Aβ_42_: 127 ± 6%; BgTx/Aβ_42_: 101 ± 9% of control). Interestingly, BgTx application was unable to completely block decreased syt1 Ab uptake mediated by supraphysiological concentration (1 μM) of Aβ_42_ (Figures 4A,B; BgTx: 89 ± 6%; Aβ_42_: 68 ± 5%; BgTx/Aβ_42_: 81 ± 4%). This indicates that the effect mediated by higher concentrations of the peptide may involve not only α7nAChRs-dependent signaling, but also other types of either pre- or postsynaptic receptors (Lauren et al., 2009; Nikolaev et al., 2009). Next, we wondered whether activation of α7nAChR is sufficient to mimic Th-induced upregulation of syt1 Ab uptake. To this end, we applied choline (Ch, 500 μM), an agonist of α7nAChR (Alkondon et al., 1997), 20 min prior to Th or control treatment. Interestingly, choline had no effect on network activity-driven syt1 Ab uptake in control cells, however it completely blocked Th-induced presynaptic activity when applied together with Th (Figure 4C; Ch: 110 ± 9%; Th: 149 ± 9%; Ch/Th: 91 ± 5% of control). This might be due to the well-known, fast desensitization of AChRs upon choline binding. To confirm this, we tested the impact of PNU120596, an allosteric modulator of α7nAChR, which has been shown to increase mean opening time of these receptors and thereby interfere with channel desensitization (Hurst et al., 2005). PNU120596 (3 μM, 1 h), had no effect on syt1 Ab uptake (Figure 4C; PNU: 114 ± 6% of control). Co-application of PNU120596 with choline fully mimicked Th-induced response (Figure 4C; PNU/Ch: 155 ± 11% of control) but PNU120596 was unable to further potentiate the effect of Th (Figure 4C; PNU/Th: 145 ± 15% of control), suggesting that endogenous Aβ might influence both channel opening and stabilization. Importantly, the Th-mediated increase in the depolarization-induced syt1 Ab uptake was completely precluded by preincubation of cells, for 30 min, with BgTx before Th application for further 1 h (Figure 4D; Th: 140 ± 7%; BgTx: 108 ± 8%; BgTx/Th: 100 ± 7% of control). Our data reveal that the Aβ-induced potentiation of presynaptic function relies on the modulation of α7nAChRs.

**Figure 5:**
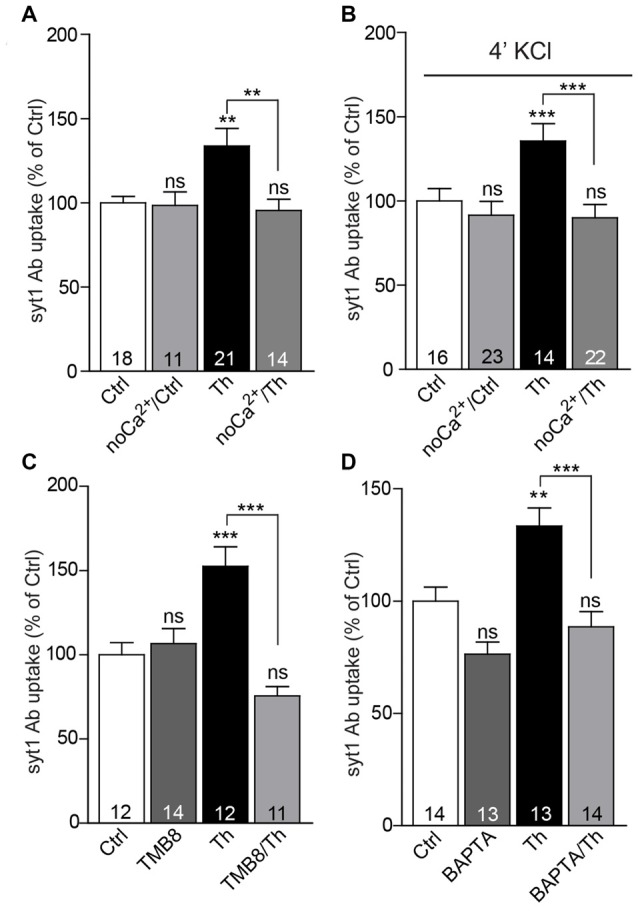
Calcium signaling via α7nAChRs is involved in the Th-induced presynaptic strengthening. **(A)** Quantification of activity-driven syt1 Ab uptake from control or Th-treated cells in the presence or absence of extracellular calcium.** (B)** The same experiment as in **(A)** but under evoked high KCl conditions. **(C)** Quantification of activity-driven syt1 Ab uptake from control vs. Th-treated cortical cells upon blockage of ryanodine receptors with TMB8. **(D)** Statistical analysis of activity-driven syt1 Ab uptake from control and Th-treated cortical neurons upon chelation of intracellular calcium by BAPTA. Numbers within columns show the number of individual cells used for analysis obtained from two independent cell culture preparations. Values are expressed as the mean ± SEM. Data were analyzed by Kruskal-Wallis test followed by Dunn’s multiple comparison test **(A)** and by one-way ANOVA with Bonferroni *post hoc* test **(B–D)**; **p < 0.01, ***p < 0.001.

One of the most prominent features of α7nACh receptors is their high Ca^2+^ conductance, which substantially contributes to synaptic Ca^2+^ signaling. To investigate whether influx of extracellular Ca^2+^ plays a role in the potentiation of SV recycling mediated by Th, we incubated both control and Th-treated cells in Ca^2+^-free medium for 1 h and subsequently performed live staining with syt1 Ab in usual imaging media containing 2 mM Ca^2+^. Incubation of cells in the Ca^2+^-free medium produced no significant effect on the network activity-driven syt1 Ab uptake (Figure 5A; Ctrl/noCa^2+^: 99 ± 8% of control) nor on the size of RP assessed by syt1 Ab uptake upon pulse application of 50 mM KCl (Figure 5B; Ctrl/noCa^2+^: 89 ± 4% of control). However, the impact of Th treatment on both network activity-driven and depolarization-induced syt1 Ab uptake was fully abolished in the Ca^2+^-free medium (Figures 5A,B; network activity driven syt1 Ab uptake: Th: 136 ± 8%; Th/noCa^2+^: 96 ± 7% of control; syt1 Ab uptake upon KCl depolarization: Th: 139 ± 8%; Th/noCa^2+^: 89 ± 4% of control).

α7nAChRs are also essentially involved in the activation of the calcium-induced calcium release pathway, which governs calcium efflux form cellular internal stores via stimulation of ryanodine receptors (RyR; Sharma and Vijayaraghavan, 2001; Dajas-Bailador et al., 2002). To test the involvement of this signaling in Th-mediated presynaptic strengthening, we applied a RyR blocker, TMB-8 (100 μM, 1 h), to the control and Th-treated cells and quantified their network activity-driven syt1 Ab uptake. The TMB-8-mediated block of Ca^2+^ release from the intracellular stores efficiently impeded Th-induced effect on the SV recycling (Figure 5C; Th: 153 ± 11%; TMB-8/Th: 76 ± 5%; TMB-8: 107 ± 9% of control). Furthermore, application of cell-permeable calcium chelator, BAPTA-AM (10 μM, 1 h), also prevented the increase in SV recycling in Th-treated cells (Figure 5D; Th: 133 ± 8%; BAPTA-AM/Th: 89 ± 7%; BAPTA-AM: 76 ± 5% of control), suggesting contribution of calcium-dependent pathways in Aβ-driven presynaptic strengthening.

### Involvement of CDK5/Calcineurin Balance in the Aβ-Mediated Regulation of SV Pools

**Figure 6:**
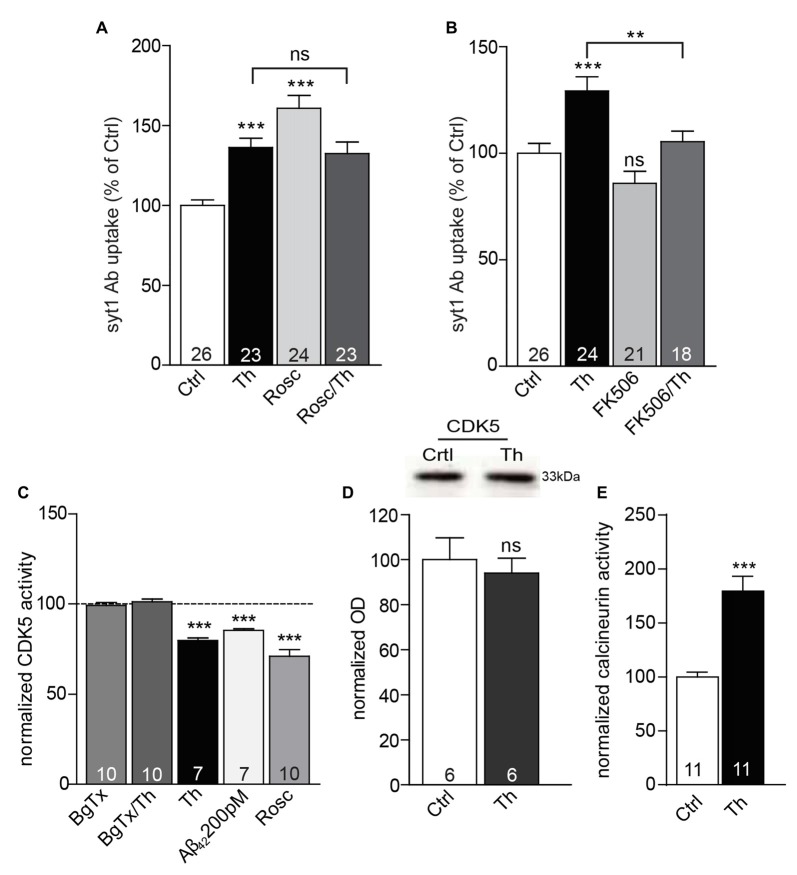
CDK5 and calcineurin are involved in Aβ-driven presynaptic plasticity. **(A)** Quantification of depolarization-induced syt1 Ab uptake from cortical neuronal cultures treated with control, Th and/or roscovitine (Rosc) to evaluate the impact of CDK5. **(B)** Statistical analysis of depolarization-induced syt1 Ab uptake from cortical cells incubated with vehicle, Th and/or FK506 to investigate the relevance of calcineurin signaling. Numbers within columns in **(A,B)** represent the number of analyzed cells obtained from three independent cell culture preparations. **(C)** CDK5 activity assay performed from cortical cultures under different conditions.** (D)** Representative Western blot as well as quantification of the total CDK5 protein levels from vehicle- or Th-treated cultures. Molecular weight is indicated in kDa. The numbers represent the number of samples from two independent primary culture preparations. **(E)** Calcineurin activity assay conducted from cortical cells incubated with vehicle or Th. Numbers within columns in **(C,E)** denote the number of independently treated and analyzed wells in a multi-well dishes obtained from three **(C)** or four **(E)** different cell culture preparations. In all graphs, values are expressed as the mean ± SEM. Statistical significance was assessed by one-way ANOVA with Bonferroni *post hoc* test **(A–C)** or Student **(D)** and Mann Whitney *t* test **(E)**; **p < 0.01, ***p < 0.001.

In the recent years, cyclin dependent kinase 5 (CDK5) and phosphatase calcineurin emerged as two major players controlling the efficacy of neurotransmitter release by modulation of the size of RP of SVs (Kim and Ryan, 2010, 2013; Marra et al., 2012). Moreover, CDK5 and calcineurin are well described targets of calcium signaling and were already previously linked to downstream signaling via α7nAChR (Patrick et al., 1999; Lee et al., 2000; Stevens et al., 2003). To address potential contribution of CDK5 and calcineurin in Aβ-mediated alternations in SV recycling, we pharmacologically blocked these enzymes and carried out syt1 Ab uptake under high KCl conditions in which all recycling vesicles undergo exocytosis and are labeled. Application of Roscovitine (1 h, 50 μM), a potent CDK5 inhibitor (Meijer et al., 1997) led to a considerable increase of syt1 Ab uptake, yet combined treatment (Th/Roscovitine) did not exert any further effect (Figure 6A; Th: 136 ± 6%; Roscovitine: 161 ± 8%; Th/Roscovitine: 132 ± 7% of control). In contrast, inhibition of calcineurin by FK506 (1 μM, 1 h) induced a significant reduction in the depolarization–induced syt1 Ab uptake in both, control and Th-treated cells (Figure 6B; Th: 129 ± 7%; FK506: 86 ± 6%; Th/FK506: 105 ± 5% of control). These results are in line with possible involvement of CDK5/calcineurin signaling in Aβ-driven regulation of SV recycling pool. To further explore this hypothesis we performed CDK5 and calcineurin activity assay. Analysis of kinase activity confirmed that cells treated with Th or 200 pM Aβ_42_, show significant reduction in CDK5 activity (Figure 6C; Th: 80 ± 1%; Aβ_42_: 85 ± 1% of control), with no change in the total protein levels (Figure 6D). Furthermore, Th-induced effect was completely prevented when α7nAChR were blocked (Figure 6C; BgTx/Th: 101 ± 2% of control). On the other hand, a phosphatase activity assay revealed significantly higher calcineurin activity in the neurons treated with Th (Figure 6E; Th: 179 ± 14% of control), corroborating that balancing the activity of these enzymes has an important role in Aβ-driven regulation of the recycling vesicles. Altogether, these results support a view that Th-mediated regulation of SV turnover involves modulation of CDK5/calcineurin phosphohomeostasis downstream of α7nAChR receptors.

## Discussion

Despite the central role of Aβ peptide in the etiopathogenesis of AD, its physiological function in the healthy brain is still poorly understood. During the past decades, Aβ emerged as a vital factor that regulates neurotransmission and studies exploring effects of physiological i.e., low-intermediate picomolar concentrations of Aβ suggested presynaptic locus of its action. In this work, we directly tested the role of Th, Aβ_40_ and Aβ_42_ species in the regulation of neurotransmitter release from the presynapse and examined the underlying cellular signaling.

### Th and Endogenous Aβ_40_ and Aβ_42_ Converge on Regulation of SV Recycling

Using quantification of syt1 Ab uptake at levels of individual synapses, we confirmed previously reported effects of Th on SV recycling in cultured rat cortical and hippocampal neurons. The authors of the original publication argued that Th effect is based on the elevation of extracellular concentrations of endogenously produced Aβ species and provided numerous, yet indirect, evidence for their claim (Abramov et al., 2009). In a following study, presynaptic strengthening was induced in the same neuronal cultures by human Aβ_40_, which, however, differs from its murine ortholog in its N-terminal sequence (Fogel et al., 2014). Thus, the presynaptic effect of endogenous Aβ_40_ and Aβ_42_ has never been explicitly demonstrated and compared. Our experiments extend previous findings and show that Th and 200 pM Aβ_40_ or Aβ_42_, in parallel experiments, exert the same effect, namely an increase in the turnover of SVs. All treatments could be efficiently blocked by inhibition of α7-nAChRs, which strongly speaks for their common mechanism of action. Interestingly, we observed smaller relative increase in the size of the recycling pool of SVs compared to the effect seen in the depolarization-driven syt1 Ab uptake assays, which might reflect differences in vesicle origin released by the chemical and electrical stimuli. Accelerated compensatory endocytosis of released SVs could also contribute to this effect and should be tested in future studies. In contrast to the previous studies that argued against any impact of physiological Aβ on the inhibitory synapses, we observed an Aβ treatment-induced increase in the network activity-driven recycling of SVs in the inhibitory synapses (Abramov et al., 2009). However, we cannot exclude that effect shown here (Figure 1F) simply reflects an increase in the overall network activity upon Th treatment and not direct regulation of release at inhibitory synapses.

### Calcium Influx via α7nAChR is Required for Aβ-Mediated Increase in SV Recycling

Involvement of α7nAChR in the modulation of neurotransmission by Aβ is controversial. The human and rodent Aβ_42_-induced memory enhancement and LTP increase were absent in the knock out for the α7 subunit of nAChR and Aβ_42_-induced LTP was also sensitive to the antagonists of α7nAChR BgTx and mecamylamine (Puzzo et al., 2008, 2011). In contrast, the presynaptic strengthening shown by imaging of activity-induced styryl dye destaining upon treatment with Th and human Aβ_40_ was not sensitive to pharmacological block of these receptors (Fogel et al., 2014). In the experiments described here, the effect of Th and picomolar concentrations of Aβ_42_ on network activity- and depolarization-driven SV recycling was fully prevented upon pretreatment with α7nAChR competitive antagonist-BgTx. In line with a requirement of the α7nAChR-mediated calcium influx for the Th-induced increase in SV recycling the effect of Th was absent upon depletion of extracellular and/or intracellular calcium and upon interference with calcium-induced calcium release from cellular internal stores. The effect of Th was also lost in the cells pretreated with choline, an agonist of α7nAChR that at high concentrations induces a rapid desensitization of the channel. Interestingly, a co-application of desensitizing concentration of choline and the allosteric modulator of α7nAChR PNU120596, known to increase opening time of agonist-bound receptor and decrease the receptor desensitization was comparable to the Th-mediated increase in SV recycling. Co-application of PNU120596 and Th did not further potentiate SV recycling suggesting that Th might affect receptor desensitization kinetics similarly as PNU120596. However, it is still unclear, whether Aβ_40_ and Aβ_42_ act as agonists or modulators of α7nAChR and what are the cofactors required for their action. While the impact of 200 pM Aβ_40_ and Aβ_42_ was hindered by pharmacological interference with α7nAChR, a blockage of these receptors did not fully prevent the effect of 1 μM Aβ_42_. This might imply that at higher concentrations Aβ_42_ acts via different cell receptors. Nevertheless, displacement measurements of Aβ_42_ and BgTx on radiolabeled α7nAChR suggested a competition of the two compounds on the same binding site with Aβ_42_ having a 4000-fold higher affinity as compared to BgTx (Wang et al., 2000b). Therefore, our result might be also explained by an incomplete blockage of α7nAChR with BgTx in the presence of 1 μM Aβ_42_.

### Endogenous Aβ Modulates Recycling SV via Modulation of CDK5 and Calcineurin Activity

Quantification of depolarization-induced syt1 Ab uptake and sypHy imaging in living neurons shown here strongly implies that Th, Aβ_40_ and Aβ_42_ control neurotransmitter release via regulation of the recycling of SVs. Recently, CDK5 and calcineurin were suggested to govern the recycling of SVs by setting a balance in the local phospho- and dephosphorylation (Kim and Ryan, 2010, 2013; Marra et al., 2012). We have shown that application of Th or Aβ_42_ leads to a rapid decrease of CDK5 activity in the cell lysates. This decrease in CDK5 activity required normal α7nAChR signaling substantiating their function upstream of CDK5 in the regulation of SV recycling by Aβ. Pharmacological inhibition of CDK5 by roscovitine mimicked Th-induced increase in the depolarization-induced syt1 Ab uptake and co-application of Th and roscovitine had no additive effect, suggesting that they share common pathways in regulation of the SV turnover. Th application increased the calcineurin activity and a pharmacological inhibition of calcineurin activity interfered with the Th-induced increase in the depolarization-induced recycling of SVs. At present, we can only speculate about signaling pathways connecting calcium influx via α7nAChR and regulation of calcineurin and CDK5 activity. Previously reported activation of calcineurin by calcium influx through α7nAChRs is compatible with our observations (Stevens et al., 2003). Cleavage of p35, an activator of CDK5, was observed upon application of micromolar Aβ_42_ and resulted in a formation of CDK5/p25 hyperactive complex (Patrick et al., 1999; Lee et al., 2000). This scenario contradicts our results that demonstrate calcium influx-induced decrease of CDK5 activity. It is possible that a slight elevation of intracellular calcium upon Aβ-mediated activation of α7nAChR has different consequences for CDK5 regulation than a massive calcium influx induced by a prolonged application of micromolar Aβ_42_.

Taken together, our data support the function of endogenous Aβ species in the regulation of neurotransmitter release. The described modulation of presynaptic function by Aβ was rapid and reversible. Moreover, a depletion of endogenous Aβ upon interference with its production and application of elevated Aβ concentrations led to a decrease in the synaptic strength, which is in accordance with previously proposed hormetic regulation of neurotransmission by endogenous Aβ (Puzzo et al., 2008; Abramov et al., 2009). Thus, our results corroborate on function of endogenous Aβ as a physiological regulator of presynaptic efficacy. Compellingly, the fast modulation of release by Aβ might act in tuning of synaptic strength at the level of individual synapses in processes of synaptic plasticity. The observation that intracellular signaling cascades, shown here to be involved in the physiological regulation of SV recycling by Aβ, are also implicated in AD pathogenesis supports the hypothesis that failure of the physiological function of Aβ in the tuning of SV recycling could impair synaptic homeostasis and initiate synaptic dysfunction leading to cognitive decline and neurodegeneration in AD.

## Author Contributions

VL and AF conceptualized the study and curated the data. VL, SF, MA-A and DA performed investigations, formal analysis, methodology development and validation. CM-V, DI, MAC provided methodologies. AF and EDG provided infrastructure and resources. VL, SF and MA-A executed visualization of data. AF supervised the study. VL, SF and AF have written original draft. All authors reviewed and edited the final manuscript.

## Conflict of Interest Statement

The authors declare that the research was conducted in the absence of any commercial or financial relationships that could be construed as a potential conflict of interest.
